# EBV Infection in XLP1 Manifested Solely by Behavioral Aggression and Effective Treatment Using Rituximab

**DOI:** 10.1155/2018/3705376

**Published:** 2018-06-07

**Authors:** Michelle M. Korah-Sedgwick, Luke A. Wall

**Affiliations:** ^1^Section of Pulmonary/Critical Care & Allergy/Immunology, Louisiana State University School of Medicine, New Orleans, 1901 Perdido Street, Suite 3205, New Orleans, LA 70112, USA; ^2^Section of Allergy/Immunology, Department of Pediatrics, Louisiana State University School of Medicine, New Orleans, Children's Hospital of New Orleans, 200 Henry Clay Avenue, New Orleans, LA 70118, USA

## Abstract

Patients with X-linked lymphoproliferative disease 1 (XLP1) are exquisitely susceptible to Epstein-Barr virus (EBV), with the first EBV infection often resulting in rapid death. In a manner not previously described, a 5-year-old patient with XLP1 presented solely with behavioral aggression, with no laboratory evidence of organ dysfunction or inflammation. Although EBV-IgM was negative, PCR confirmed the presence of EBV in both the blood and cerebrospinal fluid. MRI of the brain showed frontal lobe foci. After failure to eradicate his viremia with ganciclovir, rituximab was administered. EBV was eradicated from the blood after the second rituximab infusion and remained absent for 5 months, at which time he underwent hematopoietic stem cell transplant. Although EBV classically produces fulminant infection in patients with XLP1, this case demonstrates that EBV infection may be initially subtle. Acute change in behavior should prompt evaluation. This case also demonstrates the possible effectiveness of rituximab in the treatment of acute EBV infection.

## 1. Introduction 

X-linked lymphoproliferative disease type 1 (XLP1) is a rare primary immunodeficiency that leads to unique vulnerability to the Epstein-Barr virus (EBV). The disease is caused by germline mutations in the SH2D1A gene that encodes signaling lymphocytic activation molecule (SLAM) associated protein (SAP) [1]. SAP is expressed on T-lymphocytes, natural killer (NK) cells, and invariant natural killer T (iNKT) cells. When SAP is bound to the cytosolic SLAM, it is thought to enhance T- and B-lymphocyte proliferation, CD8+ T cell activation by antigen-presenting B cells, T-lymphocyte cytokine secretion, and B-lymphocyte antibody production [2, 3]. Additionally, SAP expression is critical for iNKT cell development; hence XLP1 patients completely lack iNKT cells [4–6]. SAP-deficient T cells fail to be activated by antigen-presenting B cells but are activated by other antigen-presenting cells. Because EBV selectively infects B cells, the T cells in patients with XLP are blind to the presence of the infection [7]. Therefore, pathogenicity is unchecked, leading to uncontrolled lymphoproliferation and severe manifestations including fulminant infectious mononucleosis and hemophagocytic lymphohistiocytosis (HLH). Fulminant infectious mononucleosis is the leading cause of mortality in these patients [8–12]. Affected children may be asymptomatic and thriving until getting infected with EBV, wherein they may quickly succumb to these conditions [13]. Additional manifestations of dysfunctional immunity in XLP1 patients include lymphoma and dysgammaglobulinemia [8, 9]. Aplastic anemia and vasculitis have also been reported [1]. Currently, the only curative treatment is hematopoietic stem cell transplantation (HSCT). This case highlights the need for a high degree of suspicion regarding EBV infection in XLP patients, as manifestations may be initially subtle. In addition, rituximab was effective in eradicating EBV in this young XLP patient with EBV-associated encephalitis.

## 2. Case Presentation 

**Figure 1:**
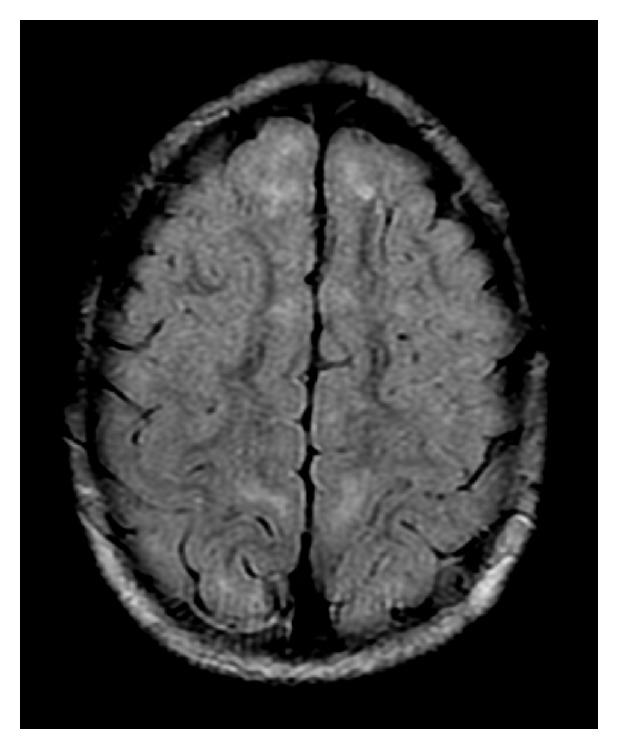
Axial T2-FLAIR MRI image demonstrating subcortical white matter hyperintensities most prominent within the frontal lobes. Image is courtesy of Dr. Jane Congeni.

**Figure 2:**
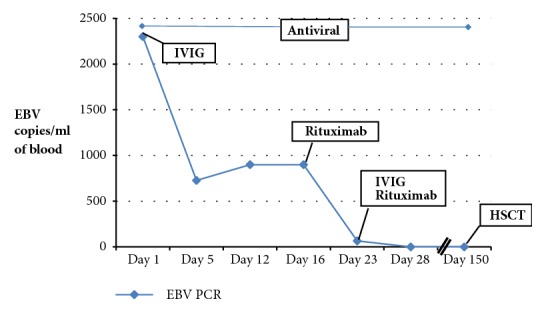
EBV copies remained at nearly 1000 per mL in peripheral blood after a 2-week treatment combination of IVIG and antivirals. EBV copies were reduced drastically with the first dose of rituximab and became undetectable following the second dose.

A 5-year-old male initially came to the attention of the immunology service at 2 years of age with a history of recurrent sinopulmonary infections and a family history of XLP1 (nonsense mutation c. 191G>A in the SH2D1A gene). Of note, his uncle with XLP1 had a history of EBV-related central nervous system (CNS) lymphoma. SAP expression was found to be absent in NK and CD8 cells, confirming the diagnosis of XLP1. NKT cells were undetectable. Profound hypogammaglobulinemia was also noted. He was monitored and maintained on monthly intravenous immunoglobulin (IVIG) infusions with only minor breakthrough sinopulmonary infections. Adherence to IVIG was suboptimal due to social reasons. The family deferred stem cell transplantation. At the age of 5 years, he presented with acute behavioral changes, manifesting as uncontrolled aggression, requiring inpatient psychiatric treatment. Basic labs including complete blood count and comprehensive metabolic panel were normal. Specifically, no cytopenias, no transaminitis, and normal inflammatory markers including C-reactive protein (CRP) and platelet count were noted. MRI of the brain showed multiple small nonenhancing foci on T2 FLAIR sequence, mostly in the frontal lobes and scattered throughout the subcortical white matter (Figure 1). The lesions remained unchanged on repeat MRI at 2 weeks and again at 4 months following presentation, suggesting that these lesions may be consistent with glial scars without acute inflammation of the brain. PCR revealed presence of EBV in the blood (2300 copies/ml). Cerebrospinal fluid (CSF) also demonstrated EBV (73 copies/ml). CSF was otherwise normal apart from only slightly elevated CSF WBC (8 cells/mm3), of which 79% were lymphocytes and 21% monocytes. Intravenous gamma globulin 1 g/kg and intravenous ganciclovir 10 mg/kg/day were started immediately. However, despite 14 days of ganciclovir treatment, EBV counts remained significantly elevated. Rituximab was administered on days 16 and 23 of admission. Prednisolone 1 mg/kg/day was given daily on days 16 through 23, and IVIG 1 g/kg was repeated on day 23. Valganciclovir 45 mg/kg/day was continued for 4 months after ganciclovir treatment. Prior to rituximab infusion, EBV counts had reached 2300 copies of EBV genome/mL of blood. Five days after the second rituximab infusion CD19+ and CD20+ lymphocytes and EBV PCR were found to be absent in the blood and remained absent for 5 months, at which time hematopoietic stem cell transplant (HSCT) was performed (Table 1 and Figure 2). IgM to EBV was undetectable at the time of presentation, and the patient never seroconverted.

## 3. Discussion 

Currently available antiviral medications and gamma globulin products are not fully effective against EBV, as seen in this case, where viral copies remained significantly elevated despite two weeks of treatment with these products. Variation in viral load is known to occur even when patients are asymptomatic, which is reflected by the fluctuation in viral copies noted in this patient during the first two weeks of treatment. The extreme vulnerability of patients with XLP makes complete viral eradication a necessity. EBV infects B-lymphocytes via the EBV receptor known as CD21 or Complement Receptor 2 (CR2). Due to the B cell-dependent pathogenicity of EBV, elimination of B-lymphocytes may currently offer the only possibility of EBV clearance. Rituximab is a humanized monoclonal antibody currently FDA-approved for several disorders, including chronic lymphocytic leukemia, some polyangiitis disorders, and rheumatoid arthritis. It is targeted against CD20, which leads to elimination of B-lymphocytes expressing CD20 through antibody-dependent cytotoxicity [14], complement-dependent cytotoxicity [14], and apoptosis [15]. After rituximab treatment, B-lymphocyte numbers typically remain reduced for approximately 6 months or longer [15]. Patients are at risk for EBV reactivation or reinfection, especially once B-lymphocytes return. Rituximab has been described as an effective EBV treatment in a few other reports of patients with XLP [16, 17]. These reports described two patients with classic EBV infection with fever and malaise and one patient with fulminant mononucleosis. In accordance with limited case reports, corticosteroids were administered along with rituximab in this case [16, 17]. While steroids might not be essential, they in theory may dampen any viral induced T cell activation, while B cells are being eliminated by rituximab. Because XLP patients are at such substantial risk for development of HLH and malignancy, an expert familiar with diagnosis and treatment of these conditions should be consulted prior to the administration of steroids. Acute psychological change without any other signs or symptoms of EBV infection in a patient with XLP is a novel clinical presentation. The patient's absence of inflammatory response and normal laboratory tests is the exact opposite of what is typically expected from an XLP patient once EBV is contracted. While the pathophysiologic rationale for this exceedingly subtle clinical presentation remains unknown, this case emphasizes that EBV infection in patients with XLP occurs on a clinical spectrum that is much wider than previously thought.

## 4. Conclusion

Patients with XLP1 have extreme vulnerability to EBV infections, with the risk of severe fulminant infectious mononucleosis, secondary malignancy, HLH, and death. Practitioners caring for patients with XLP must maintain a high index of suspicion, as infection with EBV may fall on a wide spectrum of clinical severity from immediately fatal to a smoldering infection. In the setting of a rapid and profound psychological change, XLP-related pathology of the central nervous system must be suspected, with possibilities including EBV infection and/or CNS malignancy. Normal inflammatory markers, transaminases, blood counts, and normal EBV serology cannot be relied upon to rule out the possibility of an EBV infection. Detection of EBV by PCR is essential. Rituximab offers effective viral clearance through targeted B-lymphocyte elimination and thus allows temporary treatment of EBV infection in patients with XLP.

## Figures and Tables

**Table 1:** Treatment interventions and corresponding EBV counts by PCR from blood samples.

	**Treatment**	**EBV PCR (copies/ml blood)**
**Day 1**	Ganciclovir 10mg/kg/day x 28 days IV Gamma globulin 1g/kg IV	2300

**Day 5**		727

**Day 12**		900

**Day 16**	Rituximab 375 mg/m^2^	900

**Day 23**	Rituximab 375 mg/m^2^ Gamma globulin 1g/kg IV	66

**Day 28**	Valganciclovir 45mg/kg/day x 160 days orally	0

**Day 150**	HSCT	0
